# Comparison of preoperative tramadol and pethidine on postoperative pain in cats undergoing ovariohysterectomy

**DOI:** 10.1186/s12917-014-0252-1

**Published:** 2014-10-15

**Authors:** Marina C Evangelista, Rodrigo A Silva, Larissa B Cardozo, Marcia A P Kahvegian, Thais C Rossetto, Julia M Matera, Denise T Fantoni

**Affiliations:** Department of Surgery, Faculdade de Medicina Veterinária e Zootecnia, Universidade de São Paulo, São Paulo, CEP 05508-000 Brazil

**Keywords:** Analgesia, Analgesic opioids, Cats, Meperidine, Pain, Tramadol

## Abstract

**Background:**

A variety of analgesic agents are available, and which one can be used in dogs and cats is a highly controversial issue, existing however a fear in the use of opiates due to possible adverse effects that these drugs can cause. The aim of this study was to compare the analgesic effect provided by the administration of tramadol or pethidine on early postoperative pain of cats undergoing ovariohysterectomy in a double-blind prospective study. Fourty-two animals were randomly assigned into three groups. Pet received pethidine (6 mg/kg), Tra 2 received tramadol (2 mg/kg) and Tra 4 received tramadol (4 mg/kg); all intramuscularly and associated with acepromazine (0.1 mg/kg). The efficacy of each analgesic regimen was evaluated prior to surgery (baseline - T_BL_), during surgery and 1, 3 and 6 hours after extubation with subjective pain scale, physiologic parameters, serum concentrations of glucose, cortisol and IL-6.

**Results:**

Changes in cardiovascular system were not clinically relevant. There were no significant differences in pain scores (P > 0.05) during the study, although the number of rescue analgesia was significantly higher (P < 0.05) at Pet group (5/14) than Tra 4 group (0/14), whereas in Tra 2, two animals (2/14) required additional analgesia. The serum cortisol values of Pet group were significantly higher at T_1h_ T_3h_ (P < 0.05) and T_6h_ (P < 0.01) when compared to baseline (induction), also it was noticed a significant difference among the groups at T_6h_ (Pet values were higher than Tra 2 and Tra 4; P < 0.05).

**Conclusions:**

Tramadol provided adequate analgesia and it was more effective than pethidine to at least six hours for the studied animals. At the higher dose (4 mg/kg) tramadol is probably more effective, since rescue analgesia was not necessary. No significant changes were observed physiological parameter that could contraindicate the use of these opioid in described doses, for the feline species.

## Background

There are still many reasons why veterinarians do not administer analgesic drugs to their feline patients. Some reasons cited by some authors are: lack of knowledge regarding the recognition of symptoms related to pain, fear of adverse effects and toxicity. Also lack of familiarity with current therapies probably contributes to the undertreatment of pain in companion animals [1,2]. This group of agents has been the subject of this study aiming a better understanding of the analgesic potency, as well as the adverse effects that would occasionally occur with the use of opiates in cats.

Pain assessment is difficult when objective criteria are not defined. Changes in physiological variables such as heart and respiratory rate, blood pressure, and biochemical indicators (epinephrine, norepinephrine, cortisol and β-endorphin) may be influenced by other factors like stress [3]. However, the observation of spontaneous indicative of pain behavior, combined with qualitative response to palpation of surgical wound, facilitate the evaluation of analgesia effectiveness [4]. Therefore the complete evaluation and correlation of the objective variables with the available pain scales can give a wider understanding of the background and provide a better assessment of pain.

The resulting inflammation and stress of surgery are associated with increased TNF-alpha and pro-inflammatory cytokines such as IL-1β and IL-6 [5,6]. IL-6 is a pro and anti-inflammatory cytokine, a sensitive marker of tissue injury and its intensity is directly related to the extent of surgical trauma [7–9].

Physiological signs of pain in cats are: tachypnea, tachycardia, hypertension, mydriasis, and salivation. In veterinary medicine the use of multidimensional scales that take into consideration the observation of behavior and the response to palpation of the wound is considered a good method to subjectively evaluate analgesia and classify it. Knowledge of normal behavior for the individual being evaluated is essential. There are several types of pain scales, such as descriptive, numeric rating, and visual analog scales. It is now accepted that systems that include behavior assessments and observation and interaction with the animal are most reliable. As pain is correctly identified, analgesic protocol can be used safely and appropriately [10].

Opioids are widely used in analgesic techniques to treat pain in the perioperative period [11]. They provide excellent analgesia, however have been associated with undesirable side effects such as respiratory depression, increased sedation, prolonged patient recovery and decreased gastrointestinal motility [11,12]. These effects, however, are infrequent and discreet in dogs and cats, and can be reversed by naloxone, a pure opioid antagonist [13]. The fear of adverse behavioral responses with the use of opioids, such as excitation, result in misuse by veterinarians regarding effective pain therapy in feline patients [2].

Although not classified as a pure opioid, tramadol has weak affinity for μ-receptors and is also thought to interact with noradrenergic and serotonergic systems. Tramadol does produce opioid-like behavior in cats, and mydriasis might occur [10]. Until recently, the use of tramadol in cats has been empiric, but new pharmacokinetic data [14] should lay the foundation for selecting doses for clinical evaluation [15].

Pethidine (meperidine) is also a μ-agonist opioid and has been widely used in cats. It should only be given intramuscularly or subcutaneously, as intravenous injection might produce excitement [10]. Pethidine rarely causes vomiting [16]. The main drawback of pethidine is its short duration of action. In clinical practice it performs as predicted in experimental studies, producing good analgesia for little more than 1–2 h [17–19].

The aim of this study was to evaluate the analgesia obtained with the use of pethidine compared with tramadol, both administered prior to surgery in cats undergoing elective ovariohysterectomy (OSH). Assessments of cardiovascular and respiratory effects and modulation of neuroendocrine response to pain after analgesic administration were also evaluated.

## Methods

### Animals

The study was performed on 42 healthy female domestic cats, median age 12 months (range between 6 months and 5 years), mean ± SD weight 3.0 ± 0.49 kg, undergoing OSH at the veterinary hospital of the Faculty of Veterinary Medicine and Animal Sciences, University of Sao Paulo, Brazil. All cats were judged healthy (ASA status I) based on physical examination and medical history. Only meek animals according to the opinion of the owners could enter into the study. Animals with history of attacking people, or excessive fear when manipulated were discarded. One cat was excluded from the study after biting and scratching the veterinarians who tried to restrain it.

The study was authorized by written consent of the owners and the protocol (2325/2011) was approved by the university’s ethical committee in the use of animals (CEUA).

### Anesthetic and surgical procedures

Animals were randomly assigned to one of the three groups (n =14 in each group) and received acepromazine maleate^a^ (0.1 mg/kg) associated with pethidine hydrochloride^b^ 6 mg/kg (Pet group), tramadol hydrochloride^c^ 2 mg/kg (Tra 2 group) or tramadol 4 mg/kg (Tra 4 group) intramuscularly (IM), as premedication. The observer was unawared of the treatment used.

A 22-gauge catheter was placed in a cephalic vein for drugs and fluids and anesthesia was induced with propofol^d^ (3–5 mg/kg) fifteen minutes after the premedication.

Orotracheal intubation was performed and anesthesia was manteined with isoflurane^e^ diluted in 100% of oxygen through a non-rebreathing Mapleson-F circuit. All surgical procedures were performed in different dates by the same surgeon, who was a senior teacher and the technique was similar. During surgery, animals received 5 mL/kg/h of Lactated Ringer’s solution^f^ and remained in a warm blanket.

Heart rate, electrocardiogram (lead II), respiration rate, oxyhaemoglobin saturation were monitored with the use of a multiparameter bedside monitor^g^; end tidal carbon dioxide (ETCO_2_) and end tidal isoflurane concentration (ET_iso_) were given by a gas analyzer^h^ and non-invasive arterial systolic blood pressure was measured by the vascular ultrasound doppler method.

An increase of HR or Blood pressure >15% of the previous moment would be indicative of pain, promoting an increase in 0,2% of ET_iso_.

### Pain assessment

Objective and subjective analysis were performed for the assessment of postoperative pain in the most significant moments of the experiment. The objective assessment of pain was performed through the measurement of glucose, serum cortisol and IL-6 levels, and physiological parameters (heart rate – HR, respiratory rate – RR, rectal temperature and non-invasive systolic blood pressure – SAP), measured at the following time points: T_BL_–Baseline (before premedication), T_IND_ – Shortly after induction of anesthesia (15 minutes after the premedication), T_SUR_ – Immediately after clamping of the ovarian pedicle (surgery time with higher painful stimulus), T_EXT_ – Shortly after extubation (when the animal begins to leave the state of anesthesia and regain consciousness), T_1h_ – One hour after extubation, T_3h_ – Three hours after extubation and T_6h_ – Six hours after extubation.

Subjective pain scores were assessed using a composite multidimensional pain scale [20]. The method involves assessment of behavioural indicators of pain (comfort, movement, appearance, behaviour, vocalization, feeding and wound touch) assigning a score of 0–3 for each category. Thus a score of 30 indicates maximum pain and a score of zero no pain. Pain was assessed up to 6 h after extubation. The same opioid used as the premedication was administered as analgesic rescue if pain score was 9 or above. The animals remained under supervision and assessed each hour in postoperative period, but for statistical purposes only parameters obtained at T_1h_, T_3h_ and T_6h_ were considered.

Before discharge from the hospital animals received 2,2 mg/kg flunixin^i^ subcutaneously (SC) and tramadol 2 mg/kg (PO, q 8 h) was prescribed for 3 days after surgery.

### Blood withdraw and laboratory tests

Venous blood samples (1–2 mL) were collected into non-heparinized tubes with a separator gel and clot activator from a jugular vein catheter placed after induction of anesthesia. The blood samples were taken at T_IND_, T_SUR_, T_1h_, T_3h_ and T_6h_. These samples were used partially for the immediate measurement of plasma glucose levels using a single drop of blood in a glucometer^j^. The samples were than centrifuged (2500 rpm, 10 min at 4°C) soon after collection and the serum stored at −80°C pending assay.

Cortisol concentrations were obtained by laboratory analysis of serum by radioimmunoassay method^k^ and the results were expressed in μg/mL. Interleukin-6 concentrations were obtained by the method of ELISA^l^ and results expressed in pg/mL.

### Statistical analysis

Results were recorded and data analyzed. After analysis of variance (ANOVA), Dunnett’s test was performed to compare all time points with the baseline within a group. Tukey test was applied for comparison between groups within the time points of observation. For nonparametric data, such as pain scale scores and numbers of rescue analgesia, Kruskal-Wallis test was used to identify differences within a group. For comparison among groups within the time points, Mann-Whitney test was performed. Data were analyzed by use of statistical software^m^. Values of P <0.05 were considered significant. Results are shown as mean ± standard deviation (SD).

## Results

Heart rate did not differ among the groups during the study (Table 1).

**Table 1:** **Variables (mean ± SD) of physiological parameters recorded during the study**

		**T**_**BL**_	**T**_**IND**_	**T**_**SUR**_	**T**_**EXT**_	**T**_**1h**_	**T**_**3h**_	**T**_**6h**_
	Pet	38,4 ± 16,6	32,8 ± 11,3	26,0 ± 12,2	33,4 ± 13,3	37,9 ± 12,5	38,2 ± 17,2	36,1 ± 12,3
**RR**	Tra 2	53,6 ± 8,7	34,1 ± 13,1*	34,9 ± 14,7*	33,9 ± 11,9*	38,7 ± 10,7	36,6 ± 14,6	35,7 ± 10,5*
**(mpm)**	Tra4	46,0 ± 14,4	35,2 ± 13,7	23,9 ± 4,4*	30,4 ± 10,4	39,7 ± 15,8	38,5 ± 14,9	40,6 ± 21,9
	Pet	199,2 ± 27,5	195,3 ± 41,5	173,8 ± 32,4	187,6 ± 37,1	221,3 ± 28,4	210,4 ± 35,0	195,4 ± 28,2
**HR**	Tra2	188 ± 33,5	213,8 ± 58,1	158,3 ± 19,1	206,9 ± 26,8	232,4 ± 22,8	212 ± 34,1	197,7 ± 29,9
**(bpm)**	Tra4	200 ± 14,1	196,9 ± 42,3	176,1 ± 30,8	192 ± 23,4	214 ± 33,7	193,9 ± 29,2	194,4 ± 32,7
	Pet	155 ± 5	97,1 ± 11,1^†^	112,4 ± 20,9*	137 ± 22,7	145,4 ± 37,7	129,8 ± 31,3	140,8 ± 28,9
**SAP**	Tra2	165 ± 25,5	102,5 ± 15,2^†^	111,6 ± 18,5^†^	139,5 ± 28,9	137,4 ± 50,2	153,4 ± 50,2	140,5 ± 37,7
**(mmHg)**	Tra4	180 ± 41,8	111 ± 44,3^†^	115,3 ± 34,4*	136,8 ± 35	148,7 ± 39,9	137,3 ± 34,4	151,6 ± 46,3
	Pet	38,5 ± 1,1	38,9 ± 0,5	36,6 ± 1,3^†^	-	37,2 ± 1,2	38,4 ± 0,7	38,6 ± 0,5
**Temp**	Tra2	39 ± 0,7	38,2 ± 0,7	35,4 ± 1,2^†^	-	37 ± 0,9^†^	38,1 ± 1	38,9 ± 0,3
**(°C)**	Tra4	38,4 ± 0,5	38,2 ± 0,8	36,3 ± 1,3^†^	-	37,4 ± 0,8	38,3 ± 1	38,9 ± 0,4

RR = Respiratory rate expressed in movements per minute; HR = Heart rate expressed in beats per minute; SAP = Systolic arterial pressure in milimiters of mercury; Temp = Rectal temperature in Celsius; *Value significantly different (P < 0,05) from T_BL_ for that group. ^†^Value very significantly different (P < 0,01) from T_BL_. No change among the groups was observed.

Changes in respiratory parameters were not frequent (Table 1), neither clinically relevant. The RR decreased significantly (P < 0,05) at T_IND_, T_SUR_, T_EXT_ and T_6h_ in Tra 2 group and T_SUR_ in Tra 4 group when compared to T_BL_ for these groups, although no difference among groups was recorded.

There were very significant reductions (P < 0.01) in SAP values in Pet and Tra 4 groups at T_IND_ when compared to T_BL_ and significant (P < 0.05) reduction in SAP values at T_SUR_. In Tra 2 group there was a very significant decrease (P < 0.01) at T_IND_ and at T_SUR_ compared to the baseline. It was not noticed significant increase in SAP at pedicles clamping (T_SUR_) as well as differences among the groups were not recorded as shown in Table 1.

Rectal temperature significantly decreased in all groups at T_IND_ and T_SUR_ and in group Tra2 at T_1h_.

**Figure 1:**
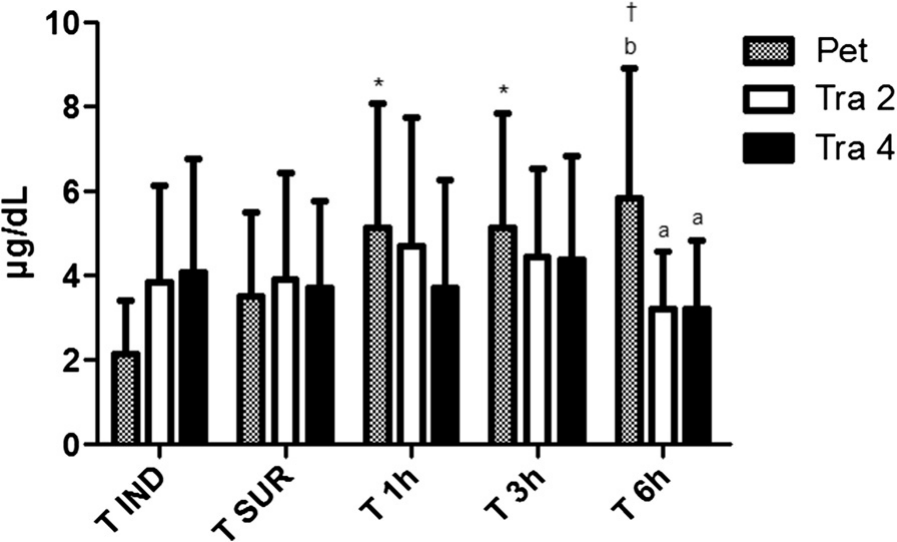
**Serum cortisol.** Serum cortisol measured in different moments of the study. *Value significantly different (p <0,05) from T_IND_ for that group. ^†^Value very significantly different (p <0,01) from T_IND_. Letters means values significantly different among the groups in each time point, as b > a.

Serum cortisol baseline values were recorded at T_IND_. Pet: 2,134 ± 1,291 μg/dL; Tra 2: 3,840 ± 2,306 μg/dL; Tra 4: 4,086 ± 2,701 μg/dL. An increase of cortisol levels in Pet group was observed in postoperative period, remaining significant higher (P < 0,05) at T_1h_ (5,160 ± 2,907 μg/dL), T_3h_ (5,154 ± 2,907 μg/dL) and very significant higher (P < 0.01) at T_6h_ (5,856 ± 3,068 μg/dL). In Pet group the values were significantly higher than in Tra 2 and Tra 4 groups at T_6h_ (Figure 1).

**Figure 2:**
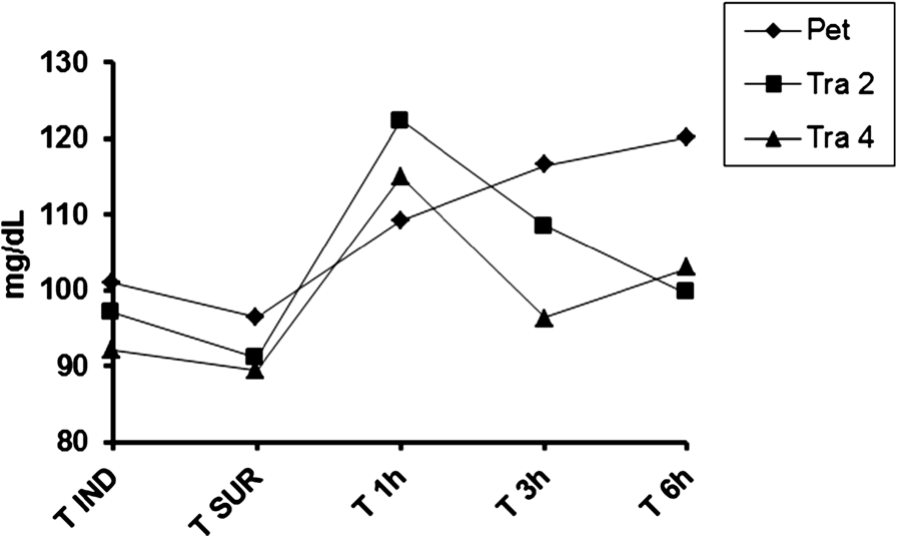
**Plasmatic glucose.** Plasmatic glucose measured in different moments of the study. No difference among the groups or among the moments was observed.

As for the values of plasma glucose (Figure 2), no significant variation occurred among the 3 groups evaluated over the moments. The mean value for all cats was 103,69 mg/dL (range of 67 to 238 mg/dL).

The interleukin-6 levels were too low and sometimes undetected by the commercial kit. The minimum sensibility of the test was 1.56 pg/mL for ELISA assay. No difference between groups was reported.

**Figure 3:**
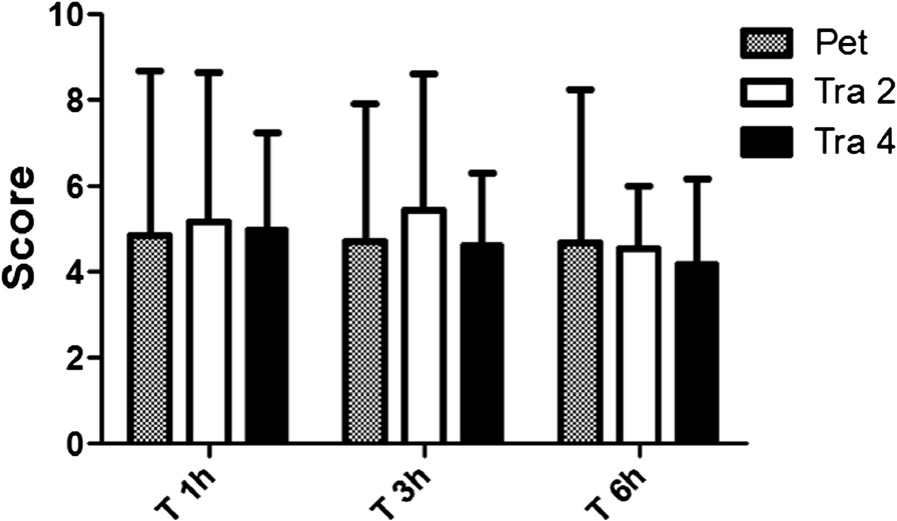
**Pain scale.** Mean ± SD pain scores according to the composite multidimensional pain scale during the study. No difference among the groups or among the moments was observed.

Regarding the composite multidimensional pain scale evaluation, in Pet group higher values of pain scores and a greater need for rescue analgesics were noted. In Tra 2 group, analgesic rescue was necessary in fewer animals and in Tra 4 group there was no need for additional analgesia during the study period, although no significant difference in pain scores was noticed within the studied period and among the groups (Figure 3).

Within Pet group, 5/14 cats received rescue analgesia. One of the cats (animal 34) received it twice. The mean ± SD time for rescue analgesia administration in pet group was 298,3 ± 152,3 minutes after premedication time (range 140–450 minutes). At Tra 2 group, 2/14 animals received additional analgesia (120 and 270 minutes after premedication, respectively). No animal required additional analgesia in Tra 4 group. Pet group number of rescues was significantly higher than Tra 4 group (P <0.05).

## Discussion

The evaluated treatments promoted adequate analgesia at postoperative period in most of cats undergoing OSH, although the animals treated with pethidine required more supplemental analgesia. By using the scale designed by Brondani et al. [20], no significant differences in pain scores within time points, or the groups were found and both agents produced adequate analgesia in most animals throughout the period of postoperative evaluation. In Pet group higher values of pain scores and a greater need for rescue analgesics were noted. In Tra 2 group analgesic rescue was necessary in only two animals and in Tra 4 group there was no need for additional analgesia during the study period. A possible explanation might be the differences in duration of action as pethidine is said to have an analgesic effect of short duration in the cat [21] and this fact might have contributed to the rescue analgesia. The administration of the same agent has controlled pain adequately thereafter and in only one animal it had again the same short duration. The assessment of pain for a longer period of time unveiled the difference of duration of the evaluated agents in the present study.

The fact that tramadol produces superior analgesia compared to that provided by pethidine might be controversial, since tramadol is an opioid with weak affinity for the μ receptor (responsible for the phenomenon of analgesia) much less intense than that of pethidine. However, the action mechanism of tramadol is not limited only to opioid receptors, it exerts its action through interactions with opioid, serotonin and adrenergic receptors. In some ways the opioid effect of tramadol is believed to be related to its metabolite, O-desmethyl-tramadol [14].

The present results corroborate previous a study result in which tramadol, at the dose of 2 mg/kg IV, did not produce any evident intraoperative cardiorespiratory side effects and no additional analgesia was required when administered as preoperative analgesic in cats undergoing surgical gonadectomy [15].

The ability of tramadol to reduce the minimum alveolar concentration (MAC) of inhalant anaesthetics has been previously reported in cats [15,22]. Acepromazine also contributes to the sparing effect of isoflurane and might have produced an antinociceptive effect as previously described [23]. In the present study, isoflurane was kept in 1 MAC and together with stable depth of anesthesia; it might indicate that adequate analgesia was obtained in all groups.

One of the cats in Tra 4 group showed signs of excitation, agressivity and proved very stressed. In previous studies, tramadol produced mild euphoria [18], but this was not deemed an undesirable attribute of the drug, seem that no other adverse effects regard to platelet aggregation, vomiting, gastrointestinal function, or biochemical values were found in other studies [21,24].

A major adverse effect of opioid analgesics is respiratory depression that is probably mediated by an effect on μ-opioid receptors [25,26]. The analgesic effect of the centrally acting synthetic opioid tramadol is thought to be mediated through both an action on these receptors and the inhibition of the reuptake of monoamines and/or stimulation of their release [14]. In the present study we evaluate the effect of opioids on the respiratory system, by monitoring RR. Our results agree with those found in literature that in anesthetized cats, 1–4 mg/kg of tramadol caused a dose-dependent depressant effect on ventilatory control [25]. Our results show that in Tra 2 group occurred a reduction in RR at induction time point (T_IND_), in Tra 2 and Tra 4 at T_SUR_ and in Tra 2 at T_EXT_ compared to T_BL_. Although no difference among the groups at these time points were noticed. At postoperative period there was no difference on the RR recorded among the groups. Although pethidine may cause respiratory depression by direct action on the respiratory centers of the brainstem [27], in the present study this effect was not observed, even in animals receiving additional administration of the agent as rescue analgesia. The decrease of the RR in the intraoperative period was predicted and is also related to the depressant action of isoflurane on respiratory centers [28]. Comparatively, both opioids proved to be safe for the respiratory system, without the occurrence of deep respiratory depression in any of the studied groups.

Regarding the effects on heart rate, our recorded data confront the investigated literature about in some aspects: in all groups, no difference in HR values during the study, when authors cite that pethidine has an augmentative effect on HR [29]. A slight decrease of HR in all studied groups occurred at T_SUR_, while an increase in HR was expected since this is the most painful moment of the surgical procedure. However, this decrease in HR in all groups during surgical procedure might be explained by the fact that animals were in surgical depth of anesthesia, in which there is no sympathetic response against surgical stress. In Tra 2 and Tra 4 groups no difference in HR was noticed throughout the study too, in agreement with the investigated literature for humans, that considers tramadol a safe opioid for cardiovascular system without causing an important bradycardia [27,30].

There were significant reductions in SAP values in all groups during anesthesia (T_IND_ and T_SUR_), which can be justified by the vasodilative effect of isoflurane in these animals [28] or by the effect of acepromazine in cats, characterized by a mild sedation, third eyelid protrusion and mild hypotension [23]. An increase in SAP at pedicles clamping could be expected as evidence of mild pain from painful stimulus caused by pedicles clamping due to limited analgesic potency of pethidine [21] and tramadol [30] at this time point. However it did not happen, furthermore isoflurane was kept in 1 MAC and together with the absence of HR increase, it could indicate that an adequate depth of anesthesia was obtained.

Concerning the rectal temperature, the values significantly decreased in all groups at T_SUR_ and in Tra 2 group remained low until T_1h_. This is justified by the depressant effect of inhaled anesthetic isoflurane on the thermoregulatory center located in the hypothalamus and also the fact that small animals are especially at risk of developing hypothermia because of their larger surface area to volume ratio compared with heavier animals [31]. In addition acepromazine is known to cause mild hypotension by vasodilation. That contributes to decreasing body temperature [23]. In the present study it was also found that opioids did not have any influence in body temperature, fact shown in all groups by the normal values of temperature measured 15 minutes after the opioid administration, at T_IND_. The literature points an opioid-related hyperthermia in cats. At doses of morphine greater than 1.0 mg/kg, cats may become hyperthermic, and pethidine at three times clinically recommended doses resulted in temperatures as high as 41.7°C [16], which was not observed in the present study.

Regarding plasma glucose levels, it was found that the three groups modulated the release of glucose as, no significant variation occurred among the 3 groups evaluated over the moments. The range of values observed is probably due to excessive handling of animals for measurements of the parameters, since some animals were naturally restless and skittish, which probably resulted in stress hyperglycemia, and not a painful background. A previous study showed that acute hyperglycemia in cats was associated with struggling during a spray bath [32].

The preoperative baseline cortisol levels, measured in animals of the three groups were slightly increased and may be correlated with the fact that most animals have been quite frightened and stressed with handling for administration of premedication and measurement of physiological parameters, since all animals participating in the experiment were healthy, so did not have an underlying disorder that alone could cause elevations in plasma cortisol levels. Baseline values of serum cortisol for all groups were close to those expected for the feline species, that range from 1,0 to 5,0 μg/dL trough the radioimmunoassay method [33,34]. It was noticed an increase in cortisol levels in Pet group after the extubation, remaining significant higher (5,160 ± 2,907 μg/dL at T1_h_; 5,154 ± 2,706 μg/dL at T_3h_ and 5,856 ± 3,068 μg/dL at T_6h)_. At T_6h_, Pet group values of serum cortisol were significant higher than Tra 2 and Tra 4. The increase in cortisol levels was expected, once abdominal surgeries promote important augments in plasma cortisol when compared to surgery on body surface area [13]. Such a rise in serum cortisol values could be an evidence of mild pain, especially in the Pet group after some time after the extubation moment, due to the termination of the analgesic effect of pethidine [21], or even as an indicative parameter of stress by physical restraint performed at the time of the measurements, however, correlating the moments of highest plasma cortisol values obtained with the subjective scale of postoperative pain, it matches the moments with higher scores in Pet group and the rescue analgesia in some of the animals. A previous study has shown no difference in plasma cortisol concentrations among cats undergoing tenotomy, onychectomy and control group, although cats included in the study had their front feet bandaged, which alone increases plasma cortisol concentrations in cats [35]. Our results matches those found in a previous study [36], where significant differences in plasma cortisol concentrations were detected among cats that underwent OSH and cats that did not receive analgesics had higher cortisol concentration than did cats without surgery and cats that received opioids after surgery. Cortisol concentration increased in response to surgical stress and pain, and this increase was diminished by the use of butorphanol, another opioid employed in feline species.

Plasmatic IL-6 concentration recorded were too low and sometimes undetected by the commercial kit, what might show that all the studied analgesics could have modulated the pain properly, while an increase in IL-6 was not noticed. Previous studies in human patients show that preemptive analgesia, commencing before surgery and continuing in the postoperative period, prevents the establishment of peripheral and central sensitization, as patients receiving preemptive analgesia exhibited less severe pain and attenuation of proinflammatory cytokine production throughout the postoperative period [37]. A control group would help identify this response, but due to ethical concerns, it was not employed in this study. Reference values for IL-6 in cats are missing in the situation, but as the surgical manipulation and inflammatory response were minimal in the group of studied animals (healthy non-obese cats), the low values found were expected.

## Conclusions

In conclusion, tramadol in both doses when administered preemptively, provided adequate analgesia and it was more effective than pethidine until seven hours after administration. Pethidine also promoted adequate analgesia, but its short duration in cats could limit the use. Tramadol, at the higher dose (4 mg/kg) is probably more effective, since rescue analgesia was not necessary to any of the evaluated animals, although one of the animals have shown signs of excitation. No significant changes were observed in physiological parameters that could contraindicate the use of these opioid in described doses, for the feline species.

Although the sample size was small, it was not detected any adverse effect of tramadol in the perioperative time, coupled with adequate analgesia produced by this agent. It may be safely employed for the treatment of mild to moderate pain in cats undergoing ovariohysterectomy.

## Endnotes

^a^Acepran 0.2%, Vetnil, São Paulo, Brazil.

^b^Dolosal, Cristália, São Paulo, Brazil.

^c^Tramadon, Cristália, São Paulo, Brazil.

^d^Propovan 1%, Cristália, São Paulo, Brazil.

^e^Isoforine, Cristália, São Paulo, Brazil.

^f^Ringer com Lactato, JP Pharmaceutical Ind., Ribeirão Preto, Brazil.

^g^DX 2010; Dixtal, São Paulo, SP, Brazil.

^h^Poet IQ, Criticare Systems Inc, Waukesha, WI.

^i^Flunixin, Chemitec Agro-Veterinária, São Paulo, Brazil.

^j^Optium Xceed, Abbott Laboratories, Brazil.

^k^Cortisol coat-a-count tubes, Siemens Medical Solution Diagnostics, Los Angeles, CA.

^l^Cat Interleukin 6 (IL-6) ELISA Kit, Cusabio, Newark, DE.

^m^Instat, GraphPad, San Diego, CA.

## Abbreviations

IL-6: Interleukin-6
IM: Intramuscular
OSH: Ovariohysterectomy
Pet: Pethidine
Tra: Tramadol
